# Changes in the neutrophil-to-lymphocyte and platelet-to-lymphocyte ratios before and after percutaneous coronary intervention and their impact on the prognosis of patients with acute coronary syndrome

**DOI:** 10.6061/clinics/2021/e2580

**Published:** 2021-07-26

**Authors:** Jianlong Sheng, Nina Liu, Fei He, Cheng Cheng, Shichun Shen, Yuting Sun

**Affiliations:** I Department of Cardiology, The Second Affiliated Hospital of Anhui Medical University, Hefei, 230601, PR China.; II Department of Endocrinology, The Second Affiliated Hospital of Anhui Medical University, Hefei, 230601, PR China.

**Keywords:** Neutrophil-to-Lymphocyte Ratio, Platelet-to-Lymphocyte Ratio, Percutaneous Coronary Intervention, Acute Coronary Syndrome

## Abstract

**OBJECTIVES:**

This study aimed to prospectively observe the changes in the neutrophil-to-lymphocyte ratio (NLR) and platelet-to-lymphocyte ratio (PLR) before and after percutaneous coronary intervention (PCI) and their impact on the prognosis of patients with acute coronary syndrome (ACS).

**METHODS:**

Blood samples from 205 patients with ACS were collected at admission and at 24h and 30 days post-PCI to observe changes in the complete blood count. The Cox multivariate regression model was used to analyze the factors influencing major adverse cardiac events (MACE) after PCI in patients with ACS. A receiver operating characteristic (ROC) curve was used to evaluate the predictive value of inflammation indicators for MACE after PCI.

**RESULTS:**

Following PCI, NLR and PLR first increased postoperatively and then decreased within 30 days after PCI. Cox multivariate regression analysis showed that NLR and PLR at 24h post-PCI and acute ST-segment elevation myocardial infarction were independent influencing factors for the incidence of MACE after PCI. The ROC curve analysis showed that the NLR at 24h post-PCI was a better predictor of the incidence of MACE. The NLR at 24h post-PCI was significantly correlated with the number and length of implanted stents and operation duration.

**CONCLUSIONS:**

After PCI, patients with ACS had an increased neutrophil proportion and NLR. The NLR at 24h post-PCI was a better predictor of the incidence of postoperative MACE.

## INTRODUCTION

Acute coronary syndrome (ACS) is a critical cardiovascular disease that can cause heart failure and arrhythmia and has a high mortality rate. Percutaneous coronary intervention (PCI) improves the prognosis of patients with ACS (1). However, among patients with ACS who receive PCI, some exhibit postoperative coronary revascularization, heart failure, cardiovascular death, and other adverse events (2,3). Inflammation plays an important role in the incidence of adverse events after PCI (4).

Previous studies have shown that the levels of inflammatory cytokines at admission are related to poor prognosis in patients with ACS (5-8). However, the results of other studies do not support this conclusion (9,10) because PCI further induces or promotes the inflammatory response (11,12). Elevated vascular inflammation may increase the risk of coronary thrombosis, restenosis in stents, unstable coronary plaque, and myocardial remodeling, thus influencing some of the postoperative benefits of PCI (13).

Theoretically, the level of inflammation after PCI may be strongly related to the prognosis after PCI. However, there are few studies regarding the impact of the levels of post-PCI inflammatory indicators on the prognosis of patients with ACS (14,15). In addition, dynamic changes in inflammatory indicators before and after PCI have rarely been reported (15,16). The neutrophil-to-lymphocyte ratio (NLR) and platelet-to-lymphocyte ratio (PLR) have been increasingly and extensively applied as inflammatory indicators in inflammation-related disease research because they are easy to use and are obtained using inexpensive tests (9,13-18). We conducted a prospective study to systematically observe the changing trends of NLR and PLR before and after PCI in patients with ACS and determine whether NLR and PLR affect the prognosis of patients undergoing PCI.

## MATERIALS AND METHODS

### Research patients

The ethical approval for this study was granted by the Ethics Committee of the Second Affiliated Hospital of the Anhui Medical University (PJ-YX2018-002). Each patient and/or his/her family provided written informed consent for participation in the study.

This study included 205 patients with ACS who underwent PCI at the Department of Cardiology of the Second Affiliated Hospital of Anhui Medical University (Anhui Province, China) between July 2018 and September 2019. The diagnostic criteria for ACS were in accordance with established guidelines (1,19). The exclusion criteria were as follows: 1) failure to follow the study protocol and complete the follow-up; 2) patients designated as having class IV heart failure according to the New York Heart Association classification before and during hospitalization, 3) patients with complications of severe infection, mixed connective tissue disease, or malignant tumors, or 4) patients receiving glucocorticoid therapy.

### Research methods

#### ACS patient grouping and PCI

In this study, patients were divided into three groups according to the ACS subtype: 1) unstable angina pectoris (UA), 2) acute non-ST-segment elevation myocardial infarction (non-STEMI), and 3) acute ST-segment elevation myocardial infarction (STEMI). Surgical indicators and PCI procedures were performed in accordance with the relevant PCI guidelines (20). The SYNTAX scores of patients with coronary lesions were calculated based on the coronary angiography results (21). PCI-related parameters, such as the number of treated coronary arteries, number of implanted stents, lengths of implanted stents, maximum pressure of balloon dilation during PCI, and operation duration, were recorded for each patient.

#### Whole blood count and NLR and PLR calculations

Peripheral venous blood samples were collected from each patient at admission, before PCI and at 24h and 30 days after PCI. All tests were performed in the laboratory of the Second Affiliated Hospital of Anhui Medical University. The complete blood count was performed using an XE-2100 automatic blood cell analyzer and matching reagents (Sysmex Corporation, Kobe, Japan). NLR was defined as the ratio of the neutrophil count to the lymphocyte count, and PLR was calculated as the ratio of the platelet count to the lymphocyte count.

#### Detection of high-sensitivity C-reactive protein and interleukin-6 after PCI

A peripheral venous blood sample was collected from each patient 24h post-PCI. High-sensitivity C-reactive protein (hsCRP) and interleukin 6 (IL-6) were detected using an AU5800 biochemical analyzer (Beckman Coulter Inc., Brea, CA, USA) and a Cobas601 biochemical analyzer (Roche Diagnostics, Indianapolis-Marion County, IN, USA), respectively.

#### MACE during follow-up

The patients were followed up via the clinic, WeChat app, telephone, or email according to their condition to observe the occurrence of major adverse cardiac events (MACE). MACE was defined as cardiovascular death, new myocardial infarction, unplanned PCI, and progression to class IV heart failure according to the New York Heart Association classification.

### Statistical analysis

SPSS19.0 software (IBM Corp., Armonk, NY, USA) was used for statistical analysis. Normally distributed data are presented as the mean±standard deviation (x̄±S), and non-normally distributed data are presented as median and interquartile range. The comparison of measurement data that was normally distributed among groups was performed by one-way analysis of variance followed by a least significant difference post-hoc test. A non-parametric test was used for the statistical analysis of non-normally distributed data or non-uniform variance among groups (Kruskal Wallis H-rank sum test). Comparison of normally distributed measurement data before and after PCI was performed using the paired sample *t*-test. The Wilcoxon signed-rank test was used to compare the parameters of non-normally distributed measurement data before and after PCI. Correlation analysis of measurement data was performed using Pearson’s correlation analysis (normally distributed data) or Spearman’s correlation analysis (non-normally distributed data). The chi-square test was used to compare the count data among the groups. A multivariate Cox regression model was used to analyze the factors influencing the incidence of MACE in patients with ACS. A receiver operating characteristic (ROC) curve was used to evaluate the predictive value of inflammation indicators for the incidence of MACE after PCI in patients with ACS. Statistical significance was set at *p*<0.05.

## RESULTS

### General clinical indicators and PCI-related indicators

A total of 205 patients with ACS (138 male and 67 female) were enrolled in this study, including 156 patients with UA, 25 patients with non-STEMI, and 24 patients with acute STEMI. Table 1 shows the comparison of the general clinical data of patients with ACS. Patients with ACS with acute STEMI were older than patients with ACS with UA and non-STEMI (*p*=0.004). No statistical differences were found among the SYNTAX scores, number of treated coronary arteries, number of implanted stents, total length of implanted stents, and maximum pressure of balloon inflation in ACS subtypes.

### Changes in blood cell components, NLR, and PLR in patients with ACS before and after PCI

In patients with ACS, the neutrophil count and proportion at 24h post-PCI were significantly elevated as compared to those prior to PCI, and reduced to pre-PCI levels 30 days after PCI. The lymphocyte count and proportion in patients with ACS were reduced at 24h post-PCI as compared to those prior to PCI, and were restored to pre-PCI levels 30 days after PCI. As a result, the variation trends in NLR and PLR were similar to the changes in neutrophil proportion (Table 2).

### Changes in blood cell components, NLR, and PLR before and after PCI in patients with different ACS subtypes

**Figure 1:**
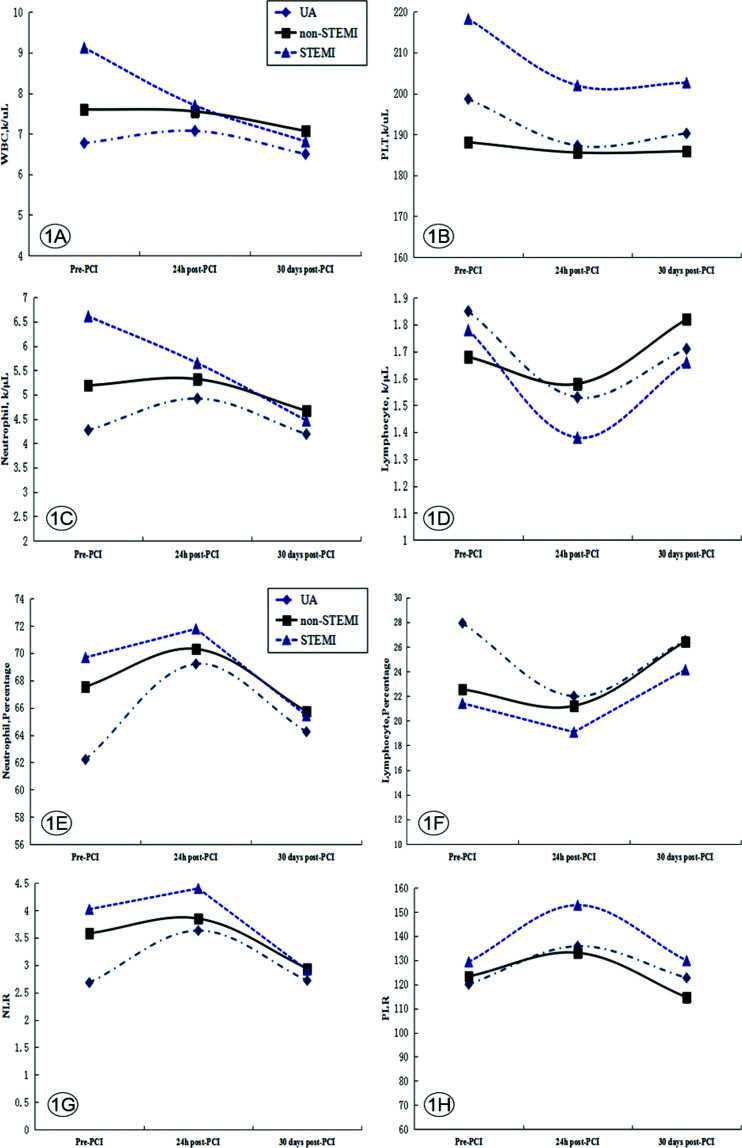
Changes in the preoperative and postoperative blood cell counts in patients with different types of ACS (n=205). Abbreviations: ACS, acute coronary syndrome; non-STEMI, acute non-ST-segment elevation myocardial infarction; NLR, neutrophil-to-lymphocyte ratio; PLR, platelet-to-lymphocyte ratio; STEMI, acute ST-segment elevation myocardial infarction; UA, unstable angina; WBC, white blood cell count.

No significant differences in the platelet count and PLR were found among the three ACS subtypes before and after PCI. In addition, the acute STEMI group showed a gradual decline in white blood cell and neutrophil counts at 24h and 30 days post-PCI relative to pre-PCI, which differed from the trend in the UA group, in which the counts first increased and subsequently decreased (Table 2 and Figure 1). The neutrophil proportion, NLR, and PLR before and after PCI showed a trend of first increasing and then decreasing in all groups. These changes suggested that PCI caused an increase in the levels of inflammatory indicators.

### Factors influencing MACE after PCI in patients with ACS

Of the 205 patients with ACS who were followed up for an average of 15 months (median) after PCI, 19 patients (9.3%) experienced MACE. The multivariate Cox regression model (entry method) was used to analyze the possible influencing factors of MACE. The results showed that the NLR at 24h post-PCI (hazard ratio [HR]=3.84, *p*=0.005), PLR at 24h post-PCI (HR=1.05, *p*=0.034), and acute STEMI (HR=4.65, *p*=0.039) were independent factors affecting the incidence of MACE after PCI. As shown in Table 3, the NLR and PLR before and 30 days after PCI had no effect on the incidence of MACE after PCI.

### ROC curve for the evaluation of predictive factors of incidence of MACE after PCI in patients with ACS

**Figure 2:**
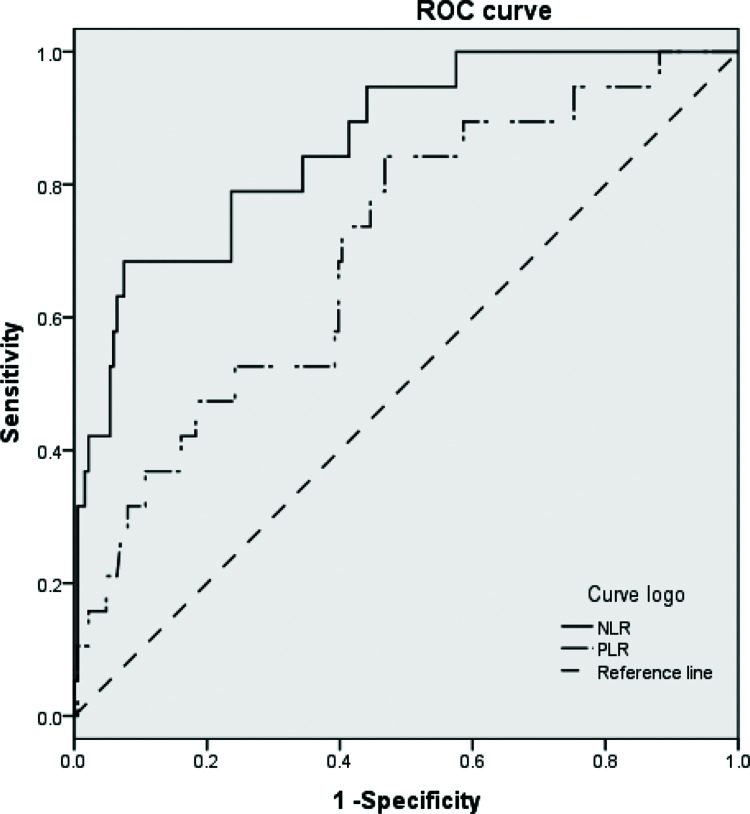
ROC curves of inflammatory biomarkers for predicting MACE after PCI. Abbreviations: PCI, percutaneous coronary intervention; MACE, major adverse cardiac events; ROC, receiver operating characteristic.

The results showed that the NLR and PLR at 24h post-PCI could be used to predict the incidence of MACE after PCI (*p*<0.01). However, the area under the curve for predicting the incidence of MACE was higher for NLR than that for PLR (0.862 *vs.* 0.703) (Table 4 and Figure 2). Moreover, the optimal cut-off point of the ROC curve for the NLR at 24h post-PCI was 3.0, with a sensitivity of 78.9% and a specificity of 67.7%, and the optimal cut-off point of the ROC curve for the PLR at 24h post-PCI was 107.01, with a sensitivity of 73.7% and a specificity of 47.8%.

### Correlation analysis of NLR and PLR

Correlation analysis showed that the NLR at 24h post-PCI significantly correlated with the number of implanted stents (*p*=0.046), length of implanted stent (*p*=0.014), operation duration (*p*=0.004), PLR (*p*<0.001), hsCRP (*p*<0.001), and IL-6 (*p*<0.001) (Table 5). There was no significant correlation between the PLR at 24h post-PCI and any of these PCI-related parameters. However, the PLR at 24h post-PCI was significantly correlated with hsCRP (*p*=0.001) and IL-6 (*p*=0.009) (Table 6).

## DISCUSSION

Inflammation plays an important role in the occurrence and development of atherosclerotic cardiovascular disease and is related to the poor prognosis of ACS (5,6,22). NLR and PLR are inflammatory indicators that have been used to assess the levels of inflammation in cardiovascular diseases in recent years (15,18). One recent study has shown that in patients with coronary heart disease and hsCRP<2.0 mg/L, the pre-PCI NLR is an influencing factor affecting the long-term prognosis of patients after PCI (23). These findings suggest that NLR may have a better predictive value in the prognosis of patients with ACS after PCI.

In this study, we found that NLR and PLR increased in patients with ACS at 24h post-PCI and then decreased over the 30 days following the operation. These dynamic trends in the NLR and PLR before and after PCI have not been reported in previous studies. The results of the Cox regression analysis showed that the NLR and PLR at 24h post-PCI were independent risk factors for MACE after PCI. However, the NLR and PLR before and 30 days after PCI had no effect on the incidence of MACE after PCI. The area under the ROC curve for NLR was greater than that for PLR, which implied that the sensitivity and specificity of NLR were higher than those of PLR.

Significant positive correlations were observed between the NLR at 24h post-PCI, the number of stents implanted during PCI, the length of the stents, and the duration of the operation. These findings suggest that the NLR after PCI is related to the complexity of the interventional procedure. After PCI, neutrophils migrate, aggregate, and adhere to arterial endothelial cells; activate platelets; and further promote the inflammatory response (24). This excessive inflammatory response may promote the progression of atherosclerotic lesions, plaque instability, atherosclerotic thrombosis (25), lack of coronary reflow after PCI (26), and increased ventricular remodeling (14), all of which can lead to post-PCI in-stent restenosis (27), acute myocardial infarction, and deterioration of cardiac function (28).

These results suggest that the NLR at 24h post-PCI is a very good indicator of the inflammatory response after PCI and has better predictive value for the prognosis of PCI than PLR. We should pay attention to the treatment and follow-up of patients with elevated inflammatory indicators after PCI, especially in patients with ACS with NLR>3.0, after PCI. Anti-inflammatory treatment with IL-1β monoclonal antibodies or intensive statin treatments in patients with coronary heart disease has been shown to reduce the incidence of MACE (29,30). Therefore, inflammation is one of the targets that requires continuous therapeutic intervention in the treatment of ACS.

This study had some limitations, such as a short follow-up period and a small sample size from a single center. The results of this study need to be confirmed by a multicenter, large-sample, long-term follow-up clinical study. Because of the lack of relevant inflammatory marker data between 24h and 30 days after PCI, precise observation of the change in the trend of inflammatory indicators after PCI was not possible.

## CONCLUSION

The neutrophil proportion and NLR of patients with ACS were elevated after PCI and were accompanied by a reduction in the lymphocyte count and proportion. In addition, compared with the PLR at 24h post-PCI, the NLR at 24h post-PCI had a better predictive value for the incidence of MACE after PCI.

## AUTHOR CONTRIBUTIONS

Sheng J designed the study design and wrote the manuscript. Liu N and He F collected and analyzed data. Cheng C and Shen S contributed to patient follow-up. Sun Y and Shen S contributed to the experimental part of this study.

## Figures and Tables

**Table 1:** Clinical characteristics of patients at baseline and post-PCI.

	UA	non-STEMI	STEMI	*p*-value
Number of patients	156	25	24	
Sex (M/F)	108/48	18/7	12/12	0.151
Age (years)	63.6±10.6	66.5±10.8	71.1±9.8*	0.004
Hypertension, n (%)	113 (72.4)	16 (64.0)	13 (54.2)	0.163
Heart failure, n (%)	8 (5.1)	1 (4.0)	2 (8.3)	0.769
Cerebrovascular disease, n (%)	26 (16.7)	3 (12.0)	5 (20.8)	0.707
Diabetes, n (%)	51 (32.7)	9 (36.0)	3 (12.5)	0.113
CKD, n (%)	8 (5.1)	1 (4.0)	2 (8.3)	0.769
Smoking, n (%)	31 (19.9)	3 (12.0)	3 (12.5)	0.480
Drinking, n (%)	18 (11.5)	2 (8.0)	1 (4.2)	0.816
Type of P2Y12 antagonist				0.120
Ticagrelor	99 (63.5)	12 (48.0)	11(45.8)	
Clopidogrel	57 (36.5)	13 (52.0)	13 (54.2)	
β-blocker treatment	125 (80.1)	20 (80.0)	20 (83.3)	0.756
ACEI/ARB treatment	112 (71.2)	19 (76.0)	18 (75.0)	0.876
Statin treatment	149 (95.5)	24 (96.0)	23 (95.8)	0.992
PPI treatment	120 (76.9)	22 (88.0)	20 (83.3)	0.635
SYNTAX score	14.3±7.6	17.0±7.1	17.1±7.7	0.071
Number of treated coronary artery (Median)	1 (1-1)	1 (1-1)	1 (1-2)	0.222
Number of stents	1 (1-2)	2 (1-2)	2 (1-2)	0.071
Total length of stents (mm)	36.0 (24.0-57.8)	52.0 (33.0-65.5)	46.0 (33.0-66.8)	0.296
Maximum pressure of balloon dilation (MPa)	1.8 (1.6-2.0)	1.8 (1.6-2.0)	2.0 (1.8-2.0)	0.770
Operation duration (min)	50.0 (36.0-66.0)	55.0 (42.5-65.5)	49.0 (40.0-60.0)	0.792

Abbreviations: PCI, percutaneous coronary intervention; UA, unstable angina; non-STEMI, acute non-ST-segment elevation myocardial infarction; STEMI, acute ST-segment elevation myocardial infarction; M, male; F, female; CKD, chronic kidney disease; ACEI, angiotensin converting enzyme inhibitors; ARB, angiotensin receptor blocker; PPI, proton-pump inhibitor; mm, millimeter; min, minute; *p<0.05, compared with the UA group.

**Table 2:** Changes in the preoperative and postoperative blood cell counts in patients with ACS undergoing PCI (n=205).

	WBC, k/μL	Platelet, k/μL	Neutrophil, k/μL	Lymphocyte, k/μL
ACS (n=205)				
Pre-PCI	7.15±2.19	199.64±58.09	4.66±1.98	1.82±0.67
24h post-PCI	7.20±1.90	188.77±55.88*	5.06±1.61*	1.52±0.57*
30 days post-PCI	6.60±1.42* ⋇	191.15±55.30*	4.28±1.13⋇	1.72±0.58* ⋇
UA (n=156)				
Pre-PCI	6.77±1.84	198.63±56.58	4.27±1.55	1.85±0.70
24h post-PCI	7.07±1.72*	187.25±54.32*	4.92±1.43*	1.53±0.57*
30 days post-PCI	6.50±1.43⋇	190.23±54.80*	4.19±1.11⋇	1.71±0.58* ⋇
non-STEMI (n=25)				
Pre-PCI	7.60±1.55	188.08±48.85	5.19±1.39Δ	1.68±0.61
24h post-PCI	7.55±1.96	185.56±48.19	5.32±1.56	1.58±0.67
30 days post-PCI	7.07±1.45	185.88±56.56	4.67±1.21⋇	1.82±0.68⋇
STEMI (n=24)				
Pre-PCI	9.12±3.46Δ	218.21±73.18	6.61±3.32Δ	1.78±0.58
24h post-PCI	7.70±2.77*	201.96±72.01	5.65±2.51	1.38±0.42*
30 days post-PCI	6.81±1.27* ⋇	202.63±58.11	4.47±1.13* ⋇	1.66±0.52⋇

	Neutrophil, percentage	Lymphocyte, percentage	NLR	PLR
ACS (n=205)				
Pre-PCI	63.74±10.43	26.50±9.02	2.95±1.83	121.52±51.72
24h post-PCI	69.65±7.69*	21.56±7.03*	3.75±1.88*	137.48±57.61*
30 days post-PCI	64.56±8.08⋇	26.22±7.21⋇	2.77±1.20⋇	122.54±52.55⋇
UA (n=156)				
Pre-PCI	62.21±9.94	27.92±8.88	2.68±1.68	120.04±52.84
24h post-PCI	69.21±7.77*	21.99±7.24*	3.63±1.68*	135.82±58.22*
30 days post-PCI	64.24±8.16* ⋇	26.51±7.16* ⋇	2.72±1.18⋇	122.68±54.38⋇
non-STEMI (n=25)				
Pre-PCI	67.54±9.54	22.55±7.35	3.58±1.87	123.29±48.92
24h post-PCI	70.31±7.75	21.21±6.96	3.85±1.75	133.10±51.72
30 days post-PCI	65.74±7.78⋇	26.42±8.58* ⋇	2.94±1.45* ⋇	114.64±47.66⋇
STEMI (n=24)				
Pre-PCI	69.69±11.65Δ	21.42±8.70Δ	4.02±2.24Δ	129.33±48.20
24h post-PCI	71.78±6.96	19.08±5.21	4.40±2.97	152.83±59.35
30 days post-PCI	65.42±8.02⋇	24.14±5.81⋇	2.91±1.06⋇	129.86±45.48

Abbreviations: ACS, acute coronary syndrome; PCI, percutaneous coronary intervention; UA, unstable angina; non-STEMI, acute non-ST-segment elevation myocardial infarction; STEMI, acute ST-segment elevation myocardial infarction; WBC, white blood cell count; NLR, neutrophil-to-lymphocyte ratio; PLR, platelet-to-lymphocyte ratio.

* *p*<0.05, compared with Pre-PCI.

⋇ *p*<0.05, compared with 24h post-PCI.

Δ *p*<0.05, compared with UA.

**Table 3:** Multiple Cox analysis of the factors predicting MACE in patients with ACS.

	B	Wald	*p*-value	HR	95% CI	
					Lower bound	Upper bound
Sex	0.82	0.65	0.419	2.27	0.31	16.64
Age	−0.07	1.31	0.253	0.93	0.82	1.05
Hypertension	2.30	2.83	0.093	10.00	0.68	146.42
Heart failure	0.08	0.00	0.959	1.08	0.05	21.23
Cerebrovascular disease	1.56	2.69	0.101	4.76	0.74	30.63
Diabetes	0.24	0.06	0.808	1.28	0.18	9.03
CKD	−2.79	2.42	0.120	0.06	0.00	2.07
Smoking	−0.34	0.04	0.840	0.71	0.03	18.53
Type of P2Y12 antagonist	−2.18	1.39	0.239	0.11	0.00	4.25
acute STEMI	1.54	4.26	0.039	4.65	1.08	20.02
SYNTAX score	0.07	1.55	0.213	1.07	0.96	1.20
NLR pre-PCI	−0.13	0.14	0.712	0.88	0.45	1.72
PLR pre-PCI	0.02	1.47	0.225	1.02	0.99	1.05
NLR 24h post-PCI	1.35	7.81	0.005	3.84	1.50	9.89
PLR 24h post-PCI	0.04	4.48	0.034	1.05	1.01	1.10
NLR 30 days post-PCI	0.68	2.14	0.143	1.97	0.80	4.86
PLR 30 days post-PCI	0.02	1.50	0.221	1.02	0.99	1.05

Abbreviations: MACE, major adverse cardiac events; ACS, acute coronary syndrome; PCI, percutaneous coronary intervention; NLR, neutrophil-to-lymphocyte ratio; PLR, platelet-to-lymphocyte ratio; STEMI, acute ST-segment elevation myocardial infarction; CKD, chronic kidney disease; CI, confidence interval.

**Table 4:** ROC curves of NLR and PLR for predicting MACE.

	Area under the curve	Standard error	*p*-value	95% CI of Area	
				Lower bound	Upper bound
NLR	0.862	0.042	<0.001	0.779	0.945
PLR	0.703	0.061	0.004	0.583	0.822

Abbreviations: MACE, major adverse cardiac events; ROC, receiver operating characteristic; NLR, neutrophil-to-lymphocyte ratio; PLR, platelet-to-lymphocyte ratio; CI, confidence interval.

**Table 5:** Correlations between NLR and age, parameters in PCI and inflammatory factors (n=205).

	r	*p*-value
Age	0.127	0.069
SYNTAX score	0.055	0.434
Number of interventional coronary arteries	0.055	0.430
Number of stents	0.140	0.046
Total length of stents (mm)	0.172	0.014
Maximum pressure of balloon dilation (kPa)	0.071	0.308
Operation duration (min)	0.199	0.004
PLR 24h post-PCI	0.616	<0.001
IL-6 24h post-PCI	0.286	<0.001
hsCRP 24h post-PCI	0.248	<0.001

Abbreviations: NLR, neutrophil-to-lymphocyte ratio; PLR, platelet-to-lymphocyte ratio; hsCRP, hypersensitive C-reactive protein; IL-6, interleukin-6.

**Table 6:** Correlations between postoperative PLR and age, parameters in PCI and inflammatory factors (n=205).

	r	*p*-value
Age	0.122	0.082
SYNTAX score	0.056	0.422
Number of treated coronary arteries	−0.007	0.919
Number of stents	0.020	0.772
Total length of stents (mm)	0.042	0.546
Maximum pressure of balloon dilation (kPa)	0.060	0.393
Operation duration (min)	0.106	0.132
NLR 24h post-PCI	0.616	<0.001
IL-6 24h post-PCI	0.183	0.009
hsCRP 24h post-PCI	0.234	0.001

Abbreviations: PLR, platelet-to-lymphocyte ratio; PCI, percutaneous coronary intervention; NLR, neutrophil-to-lymphocyte ratio; hsCRP, hypersensitive C-reactive protein; IL-6, interleukin-6.

## References

[1] Ibanez B, James S, Agewall S, Antunes MJ, Bucciarelli-Ducci C, Bueno H (2018). 2017 ESC Guidelines for the management of acute myocardial infarction in patients presenting with ST-segment elevation: The Task Force for the management of acute myocardial infarction in patients presenting with ST-segment elevation of the European Society of Cardiology (ESC). Eur Heart J.

[2] Choi DH, Kobayashi Y, Nishi T, Kim HK, Ki YJ, Kim SS (2019). Combination of Mean Platelet Volume and Neutrophil to Lymphocyte Ratio Predicts Long-Term Major Adverse Cardiovascular Events After Percutaneous Coronary Intervention. Angiology.

[3] Chen X, Meng Y, Shao M, Zhang T, Han L, Zhang W (2020). Prognostic Value of Pre-Infarction Angina Combined with Mean Platelet Volume to Lymphocyte Count Ratio for No-Reflow and Short-Term Mortality in Patients with ST-Segment Elevation Myocardial Infarction Undergoing Percutaneous Coronary Intervention. Med Sci Monit.

[4] Yang YL, Wu CH, Hsu PF, Chen SC, Huang SS, Chan WL (2020). Systemic immune-inflammation index (SII) predicted clinical outcome in patients with coronary artery disease. Eur J Clin Invest.

[5] Fanola CL, Morrow DA, Cannon CP, Jarolim P, Lukas MA, Bode C (2017). Interleukin-6 and the Risk of Adverse Outcomes in Patients After an Acute Coronary Syndrome: Observations From the SOLID-TIMI 52 (Stabilization of Plaque Using Darapladib-Thrombolysis in Myocardial Infarction 52) Trial. J Am Heart Assoc.

[6] Zhao X, Jiang L, Xu L, Tian J, Xu Y, Zhao Y (2019). Predictive value of in-hospital white blood cells count in Chinese patients with triple-vessel coronary disease. Eur J Prev Cardiol.

[7] Thomas MR, James SK, Becker RC, Himmelmann A, Katus HA, Cannon CP (2019). Prognostic impact of baseline inflammatory markers in patients with acute coronary syndromes treated with ticagrelor and clopidogrel. Eur Heart J Acute Cardiovasc Care.

[8] Wada H, Dohi T, Miyauchi K, Shitara J, Endo H, Doi S (2016). Preprocedural High-Sensitivity C-Reactive Protein Predicts Long-Term Outcome of Percutaneous Coronary Intervention. Circ J.

[9] Bressi E, Mangiacapra F, Ricottini E, Cavallari I, Colaiori I, Di Gioia G (2016). Relation of Neutrophil to Lymphocyte Ratio With Periprocedural Myocardial Damage in Patients Undergoing Elective Percutaneous Coronary Intervention. Am J Cardiol.

[10] Park JJ, Jang HJ, Oh IY, Yoon CH, Suh JW, Cho YS (2013). Prognostic value of neutrophil to lymphocyte ratio in patients presenting with ST-elevation myocardial infarction undergoing primary percutaneous coronary intervention. Am J Cardiol.

[11] Greque GV, Serrano CV, Strunz CM, Soeiro A, Santos M, Pivateli F (2016). Preprocedural statin therapy, inflammation, and myocardial injury in low-risk stable coronary artery disease patients submitted to coronary stent implantation. Catheter Cardiovasc Interv.

[12] Zhang E, Gao M, Gao J, Xiao J, Li X, Zhao H (2020). Inflammatory and Hematological Indices as Simple, Practical Severity Predictors of Microdysfunction Following Coronary Intervention: A Systematic Review and Meta-Analysis. Angiology.

[13] Bressi E, Mangiacapra F, Ricottini E, Cavallari I, Colaiori I, Di Gioia G (2018). Impact of Neutrophil-to-Lymphocyte Ratio and Platelet-to-Lymphocyte Ratio on 5-Year Clinical Outcomes of Patients with Stable Coronary Artery Disease Undergoing Elective Percutaneous Coronary Intervention. J Cardiovasc Transl Res.

[14] Hong D, Choi KH, Song YB, Lee JM, Park TK, Yang JH (2019). Prognostic implications of post-percutaneous coronary intervention neutrophil-to-lymphocyte ratio on infarct size and clinical outcomes in patients with acute myocardial infarction. Sci Rep.

[15] Kurtul A, Ornek E (2019). Platelet to Lymphocyte Ratio in Cardiovascular Diseases: A Systematic Review. Angiology.

[16] Wang Z, Liu C, Fang H (2019). Blood Cell Parameters and Predicting Coronary In-Stent Restenosis. Angiology.

[17] Gao C, Zhao D, Wang J, Liu P, Xu B (2019). Clinical significance and correlation of microRNA-21 expression and the neutrophil-lymphocyte ratio in patients with acute myocardial infarction. Clinics (Sao Paulo).

[18] Afari ME, Bhat T (2016). Neutrophil to lymphocyte ratio (NLR) and cardiovascular diseases: an update. Expert Rev Cardiovasc Ther.

[19] Roffi M, Patrono C, Collet JP, Mueller C, Valgimigli M, Andreotti F (2016). 2015 ESC Guidelines for the management of acute coronary syndromes in patients presenting without persistent ST-segment elevation: Task Force for the Management of Acute Coronary Syndromes in Patients Presenting without Persistent ST-Segment Elevation of the European Society of Cardiology (ESC). Eur Heart J.

[20] Section of Interventional Cardiology of Chinese Society of Cardiology of Chinese Medical Association; Specialty Committee on Prevention and Treatment of Thrombosis of Chinese College of Cardiovascular Physicians; Editorial Board of Chinese Journal of Cardiology (2016). [Chinese guideline for percutaneous coronary intervention(2016)]. Zhonghua Xin Xue Guan Bing Za Zhi.

[21] Sianos G, Morel MA, Kappetein AP, Morice MC, Colombo A, Dawkins K (2005). The SYNTAX Score: an angiographic tool grading the complexity of coronary artery disease. EuroIntervention.

[22] Pedicino D, Giglio AF, Ruggio A, Massaro G, D'Aiello A, Trotta F (2018). Inflammasome, T Lymphocytes and Innate-Adaptive Immunity Crosstalk: Role in Cardiovascular Disease and Therapeutic Perspectives. Thromb Haemost.

[23] Wada H, Dohi T, Miyauchi K, Nishio R, Takeuchi M, Takahashi N (2020). Neutrophil to Lymphocyte Ratio and Long-Term Cardiovascular Outcomes in Coronary Artery Disease Patients with Low High-Sensitivity C-Reactive Protein Level. Int Heart J.

[24] Warnatsch A, Ioannou M, Wang Q, Papayannopoulos V (2015). Inflammation. Neutrophil extracellular traps license macrophages for cytokine production in atherosclerosis. Science.

[25] Yilmaz M, Tenekecioglu E, Arslan B, Bekler A, Ozluk OA, Karaagac K (2015). White Blood Cell Subtypes and Neutrophil-Lymphocyte Ratio in Prediction of Coronary Thrombus Formation in Non-ST-Segment Elevated Acute Coronary Syndrome. Clin Appl Thromb Hemost.

[26] Wang Z, Ren L, Liu N, Peng J (2018). Utility of Hematological Parameters in Predicting No-Reflow Phenomenon After Primary Percutaneous Coronary Intervention in Patients With ST-Segment Elevation Myocardial Infarction. Clin Appl Thromb Hemost.

[27] Li C, Shen Y, Xu R, Dai Y, Chang S, Lu H (2019). Evaluation of Preprocedural Laboratory Parameters as Predictors of Drug-Eluting Stent Restenosis in Coronary Chronic Total Occlusion Lesions. Angiology.

[28] Xu N, Tang XF, Yao Y, Zhao X, Chen J, Gao Z (2018). Predictive value of neutrophil to lymphocyte ratio in long-term outcomes of left main and/or three-vessel disease in patients with acute myocardial infarction. Catheter Cardiovasc Interv.

[29] Ridker PM, Everett BM, Thuren T, MacFadyen JG, Chang WH, Ballantyne C (2017). Antiinflammatory Therapy with Canakinumab for Atherosclerotic Disease. N Engl J Med.

[30] Fang M, Qian Q, Zhao Z, Zhu L, Su J, Li X (2018). High-Sensitivity C-Reactive Protein Combined with Low-Density Lipoprotein Cholesterol as the Targets of Statin Therapy in Patients with Acute Coronary Syndrome. Int Heart J.

